# Role of Long Noncoding RNA 799 in the Metastasis of Cervical Cancer through Upregulation of TBL1XR1 Expression

**DOI:** 10.1016/j.omtn.2018.10.007

**Published:** 2018-10-17

**Authors:** Ling-Min Liao, Feng-Hao Zhang, Gong-Ji Yao, Su-Feng Ai, Min Zheng, Long Huang

**Affiliations:** 1 Department of Ultrasound, The Second Affiliated Hospital of Nanchang University, Nanchang, China; 2 JiangXi Key Laboratory of Clinical and Translational Cancer Research, Nanchang, China; 3 Department of Oncology, The Second Affiliated Hospital of Nanchang University, Nanchang, China; 4 Sun Yat-Sen University Cancer Center, Guangzhou, China

**Keywords:** cervical cancer, long noncoding RNA 799, TBL1XR1, miR-454-3P

## Abstract

Long noncoding RNAs (lncRNAs) are closely associated with the molecular mechanisms underlying cancer development, and it would be highly useful to study their expression and mechanisms in cervical cancer too. The current study investigated lncRNA799 expression in cervical cancer in order to determine its clinical importance in the progression of cervical cancer. lncRNA799 expression was studied in 218 cervical cancer samples. Expression of lncRNA799 was significantly higher in the cervical cancer tissue than in the adjacent normal tissue. Overexpression of lncRNA799 was found to have a significant correlation with FIGO stage, SCC-Ag level, and lymphatic metastasis, and it was also associated with poor survival. Ectopic expression of lncRNA799 promoted the metastasis of SiHa cells, whereas lncRNA799 knockdown had an inhibitory effect on metastasis. Western blot analysis demonstrated that lncRNA799 promotes the expression of transducing β-like protein 1-related protein (TBL1XR1), and that lncRNA799 and TBL1XR1 expression show strong correlation in cervical cancer. Moreover, lncRNA799 modulated the expression of TBL1XR1 by acting as a competitive endogenous RNA (ceRNA) for miR-454-3P. The results indicate that lncRNA799 could be used as a novel marker of cervical cancer prognosis. Thus, targeting the ceRNA network involving lncRNA799 could be a potential treatment strategy against cervical cancer.

## Introduction

Cervical cancer is one of the most common gynecologic malignant tumors worldwide; global statistics show that every year, about 530,000 new cases are diagnosed and 270,000 women die of cervical cancer.^1^ In recent years, increasing use of the cervical cancer vaccine and cervical smear screening has led to a decrease in the global morbidity and mortality associated with cervical cancer, but in the developing world, there has been no such decrease in cervical cancer incidence and mortality.^2^ So far, the molecular mechanism underlying the occurrence and development of cervical cancer is not entirely clear, but it would be highly useful for the identification of molecular markers, early diagnosis of cervical cancer, and the establishment of new therapeutic targets.

Long noncoding RNA (lncRNA) is an RNA molecule that plays a role in transcription; it is similar to mRNA and is commonly expressed in the eukaryotic cell genome. lncRNA was initially considered as “noise” in genome transcription; it is a byproduct of RNA polymerase II transcription and was earlier believed to have no biological function.^3^, ^4^ With microarray and the development of high-throughput sequencing technology, a large number of lncRNAs have now been discovered. As research on lncRNA function advances, a close relationship between lncRNAs and tumor development is slowly emerging.^5^, ^6^ For example, the lncRNA HOTAIR is overexpressed in breast cancers, as well as colon, pancreas, and pulmonary carcinomas, among others.^8^, ^9^, ^10^, ^11^ Moreover, high expression of HOTAIR is associated with metastasis of breast and colon cancer.^12^, ^13^, ^14^ Another identified lncRNA, MALAT-1, was reported to promote motility of cancer cells in patients with pulmonary adenocarcinoma.^15^ In addition, PRNCR1 has been found to play a role in prostate carcinogenesis.^16^ However, currently, knowledge about lncRNA expression in cervical cancer is limited. One such reported lncRNA is XLOC_010588, which was found to be an independent predictor of overall survival in cervical cancer.^17^ In addition, XLOC_008466 also acts as an oncogenic factor in cervical cancer.^18^ Another reported lncRNA is CRNDE, which was found to promote growth and metastasis of cervical cancer.^19^

Previously, we used a microarray that was developed in-house to identify lncRNA799 (NCBI: MG201855), which was highly expressed in hepatocellular carcinoma. Further, significant upregulation of lncRNA799 expression was also observed in cervical cancer. These findings indicate that lncRNA799 plays an important role in cervical cancer development, and that it may even be a new candidate tumor suppressor gene associated with cervical cancer.

In the present study, we further investigate lncRNA799 expression and its biological role in the development and progression of cervical cancer, and also delve into the potential molecular pathways that may be involved.

## Results

### High Expression of lncRNA799 in Cervical Cancer

**Figure 1:**
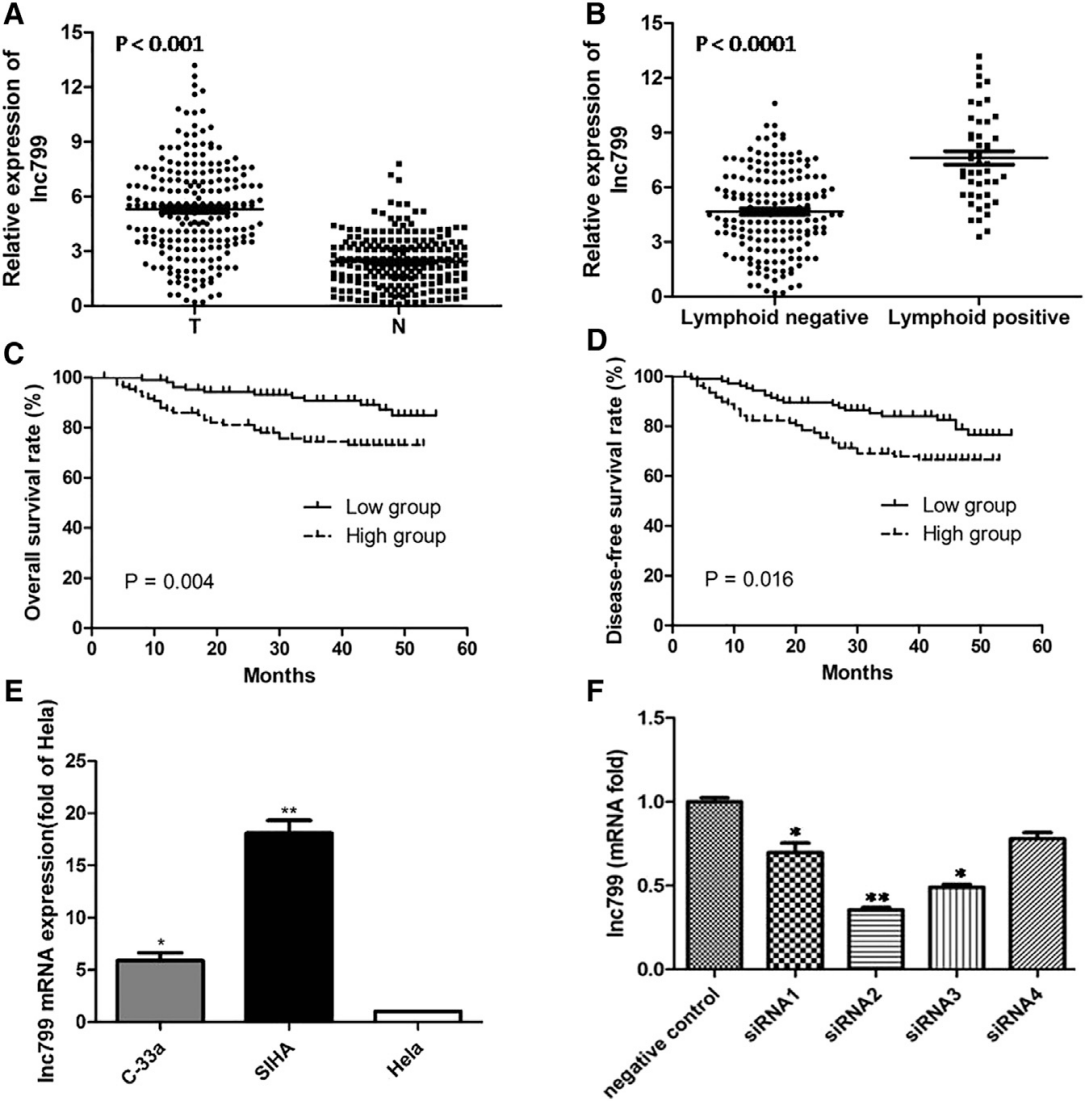
lncRNA799 Expression Was Detected by qRT-PCR (A) lncRNA799 expression was significantly upregulated in cancerous tissues compared with the corresponding normal tissues. (B) lncRNA799 expression was significantly higher in patients with lymph node metastasis. Kaplan-Meier survival curve analysis showed that patients with higher lncRNA799 expression had poorer (C) overall survival and (D) disease-free survival compared with patients with lower lncRNA799 expression. (E) lncRNA799 expression in different cervical cancer cell lines. (F) Downregulation of lncRNA799 expression in cervical cancer cell lines. Expression levels are normalized to GAPDH. Error bars represent SE. The results are from three independent experiments and reported as mean ± SD values. *p < 0.05; **p < 0.01.

qRT-PCR analysis of lncRNA799 expression in the 218 tissue samples of cervical cancer and samples of the adjacent normal tissue (control) showed significant upregulation in the cancer samples compared with the corresponding normal sample (p < 0.0001) (Figure 1A). Moreover, lncRNA799 upregulation showed a significant correlation with lymphatic metastasis (Figure 1B).

### Correlation between lncRNA799 Expression and the Clinicopathological Characteristics of Cervical Cancer

The cervical cancer samples were assigned to the high lncRNA799 expression (n = 109) and low lncRNA799 expression groups (n = 109) based on the median value of lncRNA799 expression. High lncRNA799 expression was found to be significantly correlated with International Federation of Gynecology and Obstetrics (FIGO) stage (p = 0.014), SCC-Ag level (p = 0.015), and lymphatic metastasis (p < 0.0001), but it was not correlated with age, degree of differentiation of the tumor, size of the tumor, depth of tumor invasion, or invasion of the uterine corpus (Table 1).

**Table 1:** Association of lncRNA799 Expression with the Clinicopathological Features of Cervical Cancer

Clinicopathological Features	No. of Cases	lncRNA799 Expression				p Value
		Low	%	High	%	
**Age (Years)**						

≤35	91	43	47.3	48	52.7	0.492
>35	127	66	52.0	61	48.0	

**FIGO Stage**						

IB1	155	86	55.5	69	44.5	0.014*
IB2-IIA	42	18	42.9	24	57.1	
IIb	21	5	23.8	16	76.2	

**Degree of Differentiation**						

Well	11	6	54.5	5	45.5	0.648
Moderate	134	70	52.2	64	47.8	
Poor	70	32	45.7	38	54.3	

**SCC-Ag (μg/L)**						

≤4	168	77	45.8	91	54.2	0.015*
>4	25	18	72.0	7	28.0	

**Tumor Diameter (cm)**						

≤4	158	77	48.7	81	51.3	0.958
>4	60	29	48.3	31	51.7	

**Depth of Cervical Invasion**						

<2/3	111	58	52.3	53	47.7	0.689
≥2/3	101	50	49.5	51	50.5	

**Uterine Corpus Invasion**						

Negative	69	30	43.5	39	56.5	0.205
Positive	146	77	52.7	69	47.3	

**Lymphoid Node Invasion**						

Negative	171	100	58.5	71	41.5	<0.0001*
Positive	47	9	19.1	38	80.9	

Pearson chi-square test was used for analysis. *The p values indicate significant differences.

### Correlation of lncRNA799 Expression with Cervical Cancer Prognosis

As expected, high lncRNA799 expression was associated with significantly lower overall survival (p = 0.004), as well as disease-free survival (p = 0.016), than low lncRNA799 expression (Figures 1C and 1D).

### *In Vitro* Promotion of Cell Metastasis in Cervical Carcinoma Cells by lncRNA799

**Figure 2:**
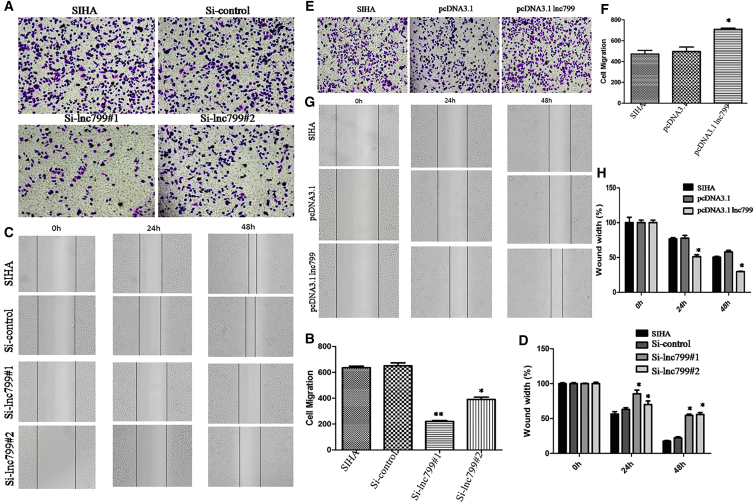
lncRNA799 Promotes the *In Vitro* Metastasis of Cervical Cancer Cells SiHa cells were transiently transfected with lncRNA799 siRNA or an lncRNA799 expression construct. Cell viability was examined using the wound healing and invasion assays. Silencing of lncRNA799 significantly inhibited the metastasis of SiHa cells compared with the control (A and B, invasion assay; C and D, wound healing assay). Overexpression of lncRNA799 significantly promoted the metastasis of SiHa cells (E and F, invasion assay; G and H, wound healing assay). The results are from three independent experiments and reported as the mean ± SD values. *p < 0.05; **p < 0.01.

**Figure 3:**
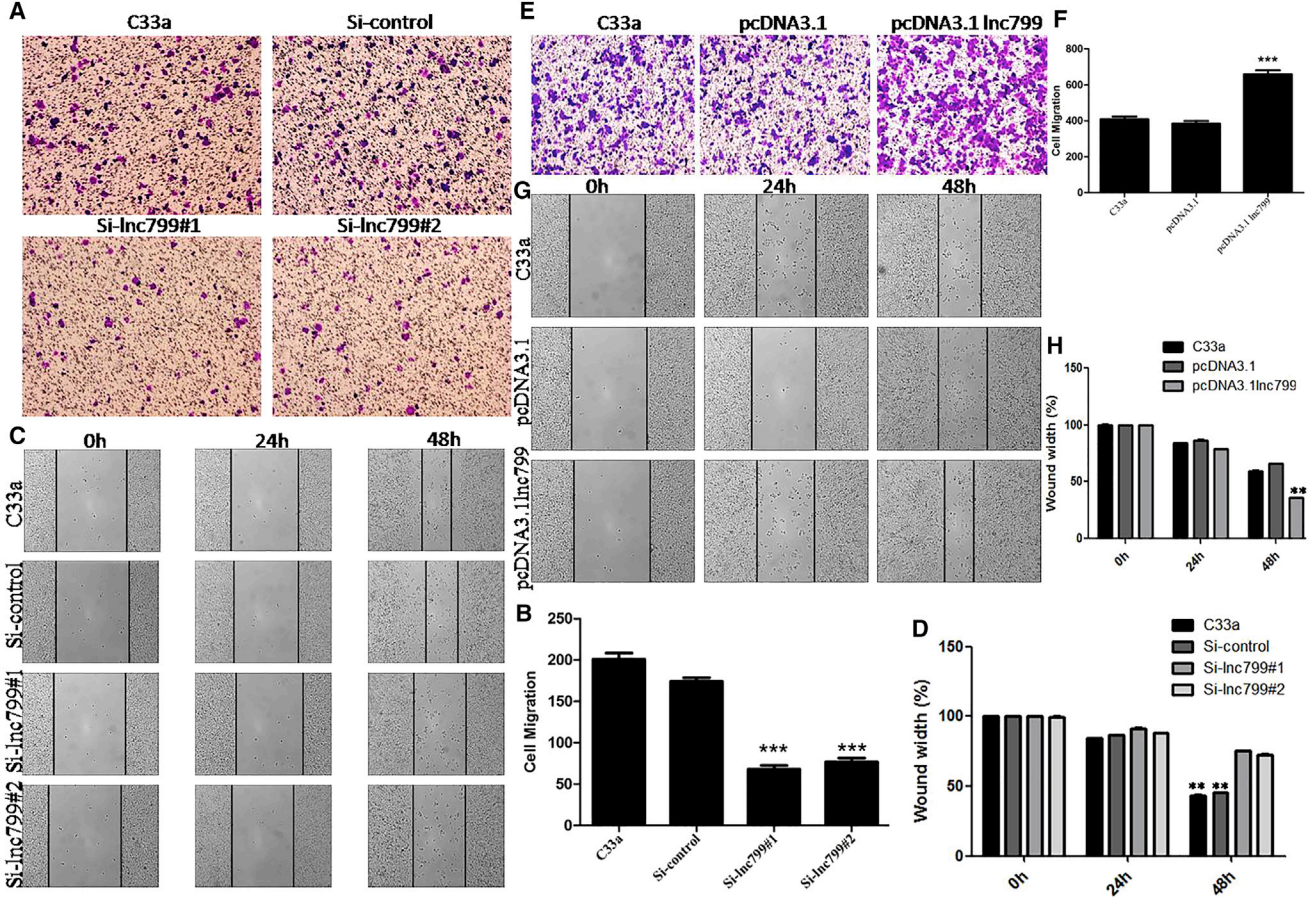
lncRNA799 Promotes the *In Vitro* Metastasis of Cervical Cancer Cells C33a cells were transiently transfected with lncRNA799 siRNA or an lncRNA799 expression construct. Cell viability was examined using the wound healing and invasion assays. Silencing of lncRNA799 significantly inhibited the metastasis of C33a cells compared with the control (A and B, invasion assay; C and D, wound healing assay). Overexpression of lncRNA799 significantly promoted the metastasis of C33a cells (E and F, invasion assay; G and H, wound healing assay). The results are from three independent experiments and reported as the mean ± SD values. **p < 0.01; ***p < 0.001.

**Figure 4:**
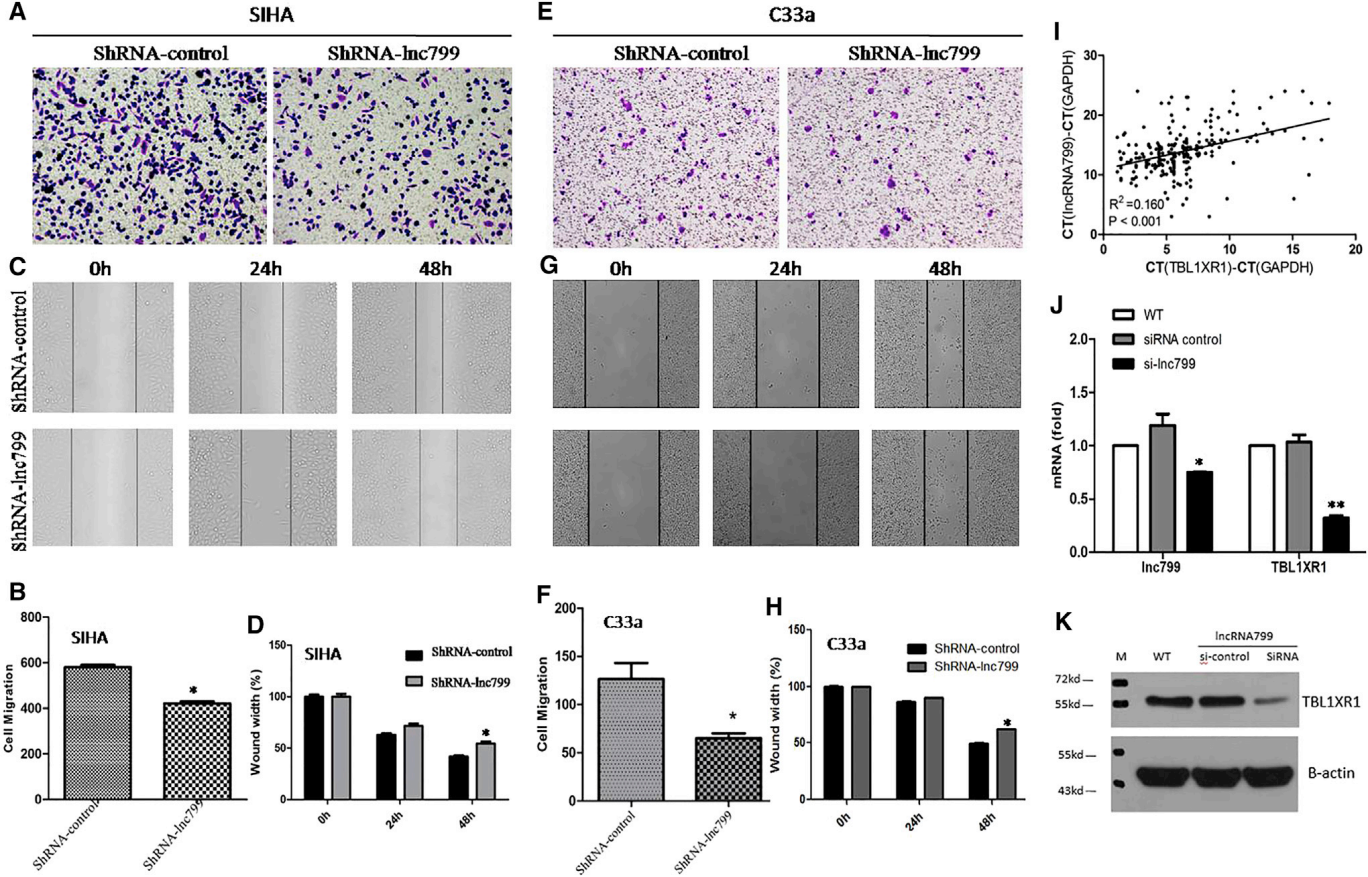
Cell Viability Was Examined Using the Wound Healing Assay and Invasion Assay in Stable Cell Lines Expressing lncRNA799 shRNA Knockdown of lncRNA799 significantly inhibited metastasis of SiHa (A and B, invasion assay; C and D, wound healing assay) and C33a (E and F, invasion assay; G and H, wound healing assay) cells compared with the control cells. (I) The lncRNA799 and TBL1XR1 expression level were measured by real-time qRT-PCR, and the respective ΔCt values (of both lncRNA799 and TBL1XR1) normalized to GAPDH were subjected to Pearson correlation analysis (r^2^ = 0.160; p < 0.001). Downregulation of lncRNA799 significantly decreased the expression of TBL1XR1 mRNA (J) and protein (K). The results are from three independent experiments and reported as mean ± SD values. *p < 0.05; **p < 0.01.

Because lncRNA799 expression showed a significant correlation with lymphatic metastasis (p < 0.0001), we next tested the hypothesis that lncRNA799 plays a regulatory role in cervical cancer metastasis in the cervical cancer cell line SiHa, which is known to express high levels of lncRNA799 (Figure 1E). lncRNA799 was knocked down by small interfering RNAs (siRNAs) (Figure 1F), and lncRNA799 overexpression was induced by transfection of SiHa and C33a cells with pCDNA3.1-lncRNA799. Cell viability was examined using the wound healing and invasion assays. Knockdown of lncRNA799 resulted in significant inhibition of SiHa (invasion assay: Figures 2A and 2B; wound healing assay: Figures 2C and 2D) and C33a (invasion assay: Figures 3A and 3B; wound healing assay: Figures 3C and 3D) cell metastasis in comparison with the control cells. In contrast, overexpression of lncRNA799 significantly promoted the metastasis of SiHa (invasion assay: Figures 2E and 2F; wound healing assay: Figures 2G and 2H) and C33a cells (invasion assay: Figures 3E and 3F; wound healing assay: Figures 3G and 3H). Induction of cell metastasis by lncRNA799 was also studied in a stable cell line expressing lncRNA799 shRNAs. As shown in Figure 4, downregulation of lncRNA799 expression in the SiHa (Figures 4A–4D) and C33a (Figures 4E–4H) cell lines significantly inhibited metastasis. Based on these findings, it seems that lncRNA799 may promote the *in vitro* metastasis of cervical cancer cells.

### Upregulation of TBL1XR1 Expression by lncRNA799

Next, we investigated the molecular pathways associated with lncRNA799-induced metastasis of cervical cancer cells. We targeted transducing β-like protein 1-related protein (TBL1XR1), which is an effector molecule present downstream of lncRNA799. TBL1XR1 mRNA and lncRNA799 expression was found to be significantly correlated in each cervical cancer tissue sample (r^2^ = 0.160; p < 0.001; Figure 4I). Further, silencing of lncRNA799 expression resulted in a significant decrease in the mRNA (Figure 4J) and protein expression (Figure 4K) of TBL1XR1 in SiHa cells. These findings indicate that lncRNA799 has a promotive effect on TBL1XR1 expression.

### lncRNA799-Stimulated Metastasis of Cervical Cancer Cells through Upregulation of TBL1XR1 Expression

**Figure 5:**
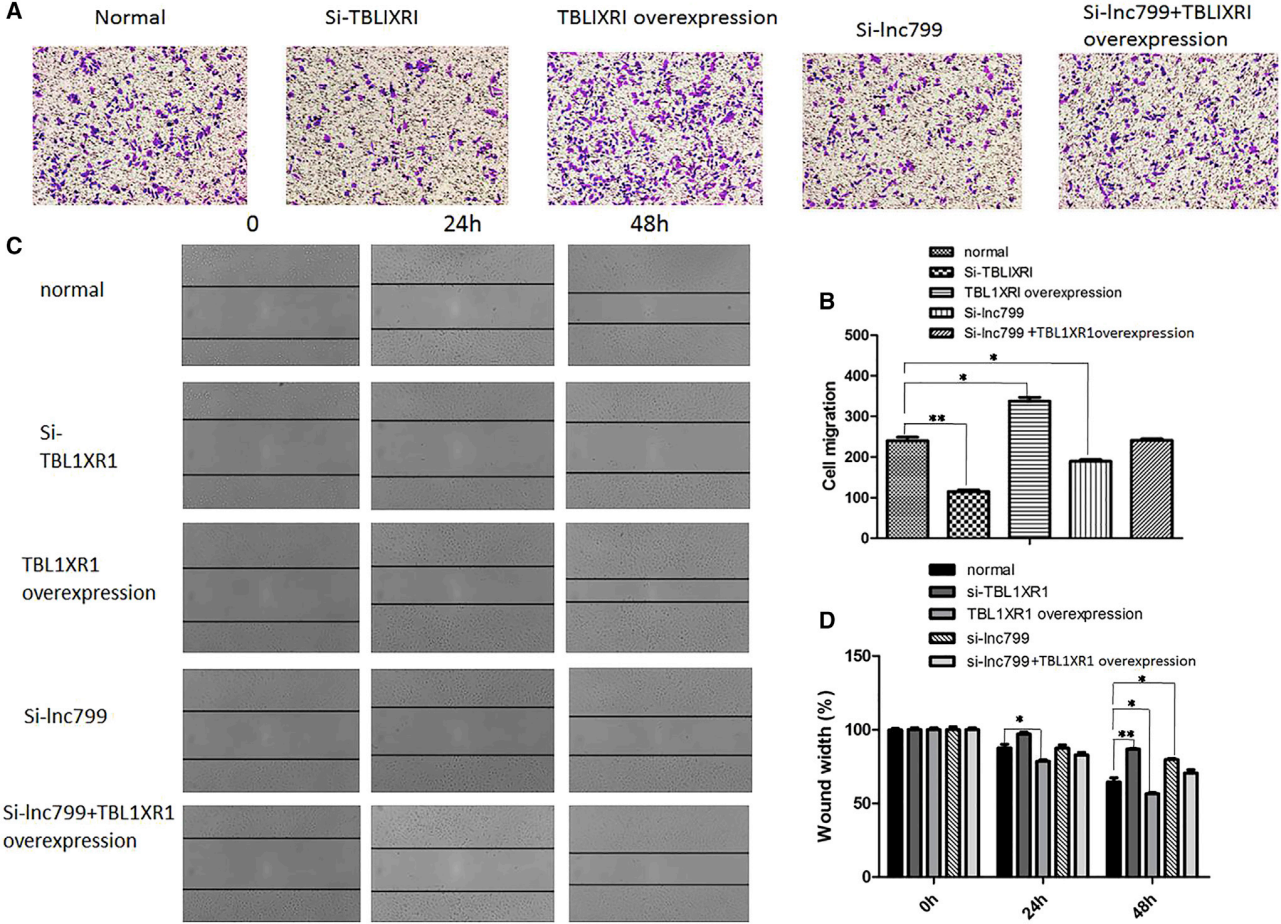
lncRNA 799 Promotes Metastasis through Upregulation of TBL1XR1 lncRNA799 promotes metastasis through upregulation of TBL1XR1 (A and B, invasion assay; C and D, wound healing assay). TBL1XR1 expression in SiHa cells was silenced by siRNA transfection and overexpressed by transfection with pCDNA3.1-TBL1XR1. TBL1XR1 silencing significantly inhibited the metastasis of SiHa cells compared with the control. Overexpression of lncRNA799 significantly promoted the metastasis of SiHa cells. Concomitant overexpression of TBL1XR1 and downregulation of lncRNA799 significantly enhanced the metastatic ability of SiHa cells compared with only downregulation of lncRNA799. The results are from three independent experiments and reported as mean ± SD values. *p < 0.05; **p < 0.01.

To investigate whether lncRNA799 promotes metastasis through upregulation of TBL1XR1 expression, TBL1XR1 was knocked down by siRNAs and overexpressed by transfection of SiHa cells with pCDNA3.1-TBL1XR1. Cell viability was examined using the wound healing (Figures 5A and 5B) and invasion assays (Figures 5C and 5D). Compared with the control cells, knockdown of TBL1XR1 in SiHa cells resulted in significant inhibition of metastasis. In contrast, overexpression of TBL1XR1 significantly promoted the metastasis of SiHa cells. Furthermore, compared with only downregulation of lncRNA799, concomitant overexpression of TBL1XR1 and downregulation of lncRNA799 significantly increased the metastatic ability of SiHa cells. These findings imply that lncRNA799 promotes metastasis in cervical cancer cells through upregulation of TBL1XR1 expression.

### Role of lncRNA799 as a Competitive Endogenous RNA for miR-454-3P in Cervical Cancer Cells

**Figure 6:**
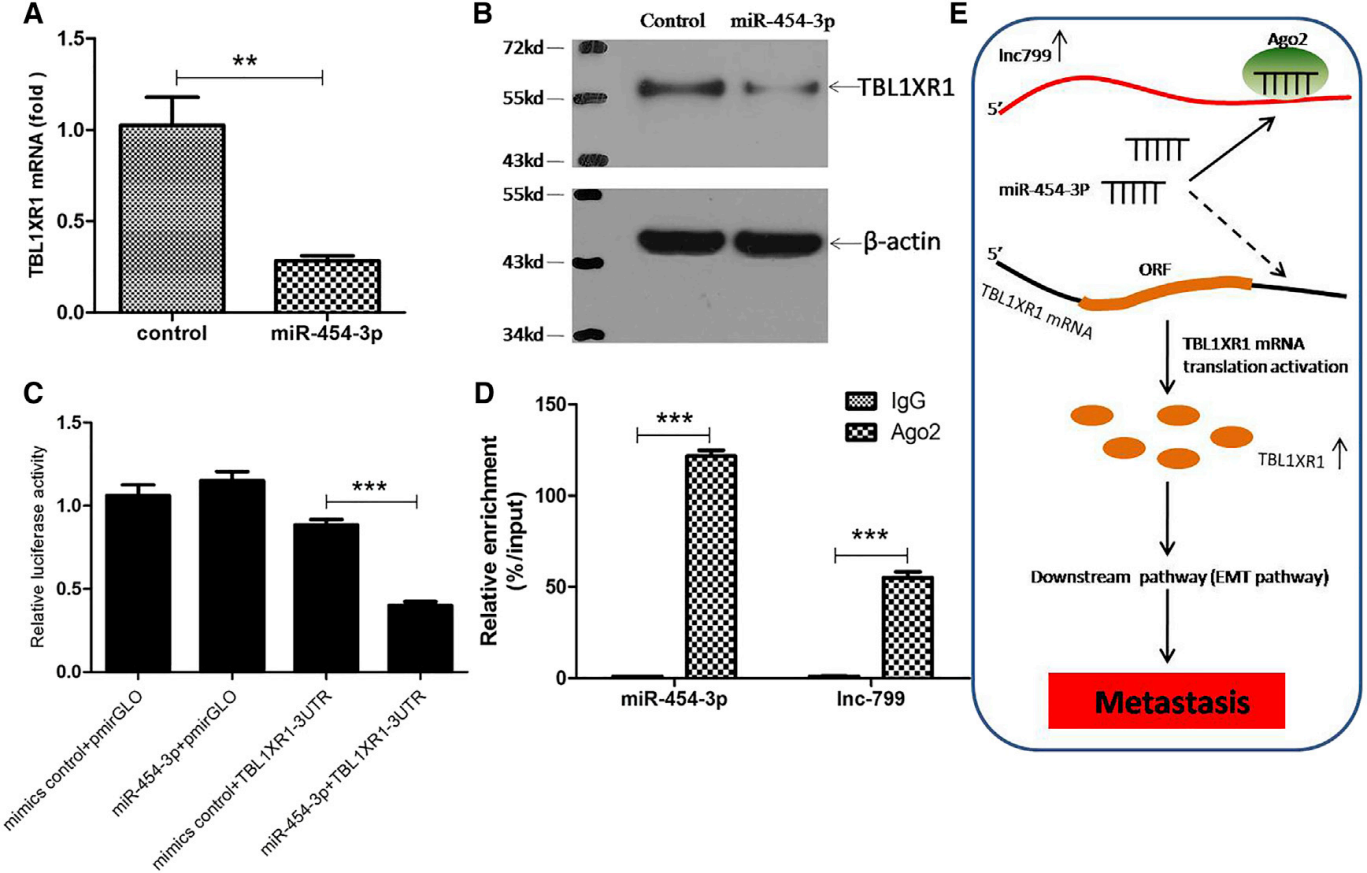
lncRNA799 Serves as a Sponge for miR-454-3p Transfection of miR-454-3p significantly decreased the expression of TBL1XR1 mRNA (A) and protein (B) expression in comparison with the control cells. (C) Luciferase activity was detected using the dual-luciferase assay. The results showed a decrease in luciferase activity in cells transfected with TBL1XR1-3UTR and miR-454-3p. Co-transfection of pmirGLO and miR-454-3p had no effect on reporter activity. (D) RNA immunoprecipitation with a monoclonal anti-Ago2 antibody was used to assess endogenous Ago2 binding to RNA; IgG was used as the control. The levels of lncRNA799 and miR-454-3p were determined by qRT-PCR and presented as fold enrichment in Ago2 relative to input. (E) Schematic of the proposed mechanism of action of lncRNA799 in cervical cancer cells. lncRNA799 acted as a ceRNA to “sponge” miR-454-3p and upregulate the expression of TBL1XR1, which promoted cellular invasion and metastasis in cervical cancer cells (**p < 0.01; ***p < 0.001).

Bioinformatics analysis using the RNA-hybrid assay revealed that the 84–92 nt site of lncRNA799 could bind miR-454-3p. Further, with the miRDB software, it was predicted that the 1,294–1,300 nt site of TBL1XR1 3ʹ UTR can bind miR-454-3p. Thus, lncRNA799 might act as a sponge for miR-454-3p. To confirm the predicted miR-454-3p target site within lncRNA799, SiHa cells were transfected with miR-454-3p, and TBL1XR1 expression was determined by qRT-PCR and western blot analyses. miR-454-3p-transfected SiHa cells showed a significant reduction in TBL1XR1 mRNA (Figure 6A) and protein (Figure 6B) expression compared with the control cells. Additionally, dual-luciferase assays with luciferase vectors constructed for TBL1XR1 3ʹ UTR (the miR-454-3p-binding motif) showed that transfection of the vector along with miR-454-3p or an miR-454-3p mimic (as the control) resulted in a significant reduction in luciferase activity (Figure 6C); however, this decrease was not observed with the empty vector pmirGLO.

To determine whether endogenous lncRNA799 was a component of microRNA (miRNA)-containing ribonucleoprotein complexes, we conducted RNA immunoprecipitation with an Ago2 antibody in SiHa cell extracts. lncRNA799 and miR-454-3p were found to be enriched in Ago2-containing miRNA-containing ribonucleoprotein complexes in comparison with the controls (immunoglobulin G [IgG] immunoprecipitates); this indicates that the Ago2 protein directly binds to lncRNA799 and miR-454-3p in cervical cancer cells (Figure 6D). These results show that lncRNA799 was physically present in Ago2-containing miRNA-containing ribonucleoprotein complexes in association with miR-454-3p.

## Discussion

To the best of our knowledge, the present study is the first to report upregulation of lncRNA799 expression in cervical cancer. In addition to this, we have also shown that increased lncRNA799 expression is correlated with lymphatic metastasis, and that TBL1XR1 is involved in the underlying molecular mechanism. These results indicate that lncRNA799 may play a role in the occurrence and growth of cervical cancer via regulation of TBL1RX1 expression.

In the early stages, cervical cancer is typically treated with radical hysterectomy combined with lymphadenectomy or chemoradiation. Additionally, patients with metastasis of the lymph node require additional adjuvant therapy. The FIGO guidelines classify patients as high risk or intermediate risk, based on various pathological risk factors, such as pelvic lymph node invasion, microscopic parametrical invasion, resection margins that are tumor positive, and invasion into the deep stromal region. The need for adjuvant therapy is determined based on this classification. Pelvic lymph node metastasis is associated with the highest risk for recurrence, and patients with this risk factor are usually given adjuvant therapy. However, sentinel lymph node biopsy and other imaging methods in use have low accuracy for the detection of micrometastases. Considering these current limitations in the diagnosis and management of cervical cancer, lncRNA799 could function as a predictor of metastasis to the pelvic lymph nodes in patients with cervical cancer.

With regard to the mechanisms by which lncRNA799 promotes lymphatic metastasis, the present findings show that lncRNA799 promoted metastasis by upregulating TBL1XR1 expression. Further, TBL1XR1 mRNA and lncRNA799 expression were found to be correlated; this confirms the regulatory effect of lncRNA799 on TBL1XR1 expression. TBL1XR1, which was recently discovered, has been found to be an important mediator in the synergistic function of corepressors, which play a role in essential functions at the cellular level, such as growth and antiapoptotic and inflammatory mechanisms.^20^, ^21^ Moreover, TBL1XR1 expression is upregulated in human primary lung squamous cell carcinoma, breast cancer, and colon cancer; this indicates that TBL1XR1 plays a role in the progression of cancers.^22^, ^23^ Interestingly, knockdown of TBL1XR1 resulted in a significant decrease in invasion in the case of head and neck squamous cell carcinoma; thus, TBL1XR1 is probably essential for tumorigenic and metastatic mechanisms.^24^ Thus, the TBL1XR1 protein could be a marker of cervical cancer prognosis and play a key role in cervical cancer invasion and metastasis.^25^

In the present study, lncRNA799 overexpression resulted in a significant increase in TBL1XR1 expression and increase in cervical cancer metastasis, whereas lncRNA799 knockdown had the opposite effect. Further, lncRNA799 was found to bind directly to miR-454-3p. Thus, our data indicate that lncRNA799 plays the role of an endogenous sponge, or competitive endogenous RNA (ceRNA), to control the availability of miR-454-3p for its target gene. ceRNAs, which can be artificial or natural, function as competitive inhibitors by sequestering miRNAs and saturating the miRNA pool, thereby suppressing the binding of miRNAs to their targets.^26^, ^27^ Interestingly, our findings indicated an endogenous interaction between lncRNA799 and miR-454-3p via co-immunoprecipitation with the Ago2 protein in cervical cancer cells. The role of lncRNAs as ceRNAs has been reported in recent studies. Similar to lncRNA700, lncRNA HOTAIR was found to promote gastric cancer progression by acting as a ceRNA for miR-331-3p and thereby increasing HER2 expression.^28^ Further, the lncRNA BARD1 9’L was found to influence BARD1 mRNA expression by acting as a sponge for miR-203 and miR-101.^29^ Although the research on ceRNAs is in its initial stages, emerging evidence indicates that this is a new discovery in posttranscriptional gene regulation. ceRNAs can have profound implications for cancer initiation and progression.^30^ Further, given the huge number of lncRNAs known, a systematic investigation into their function is highly warranted.

In conclusion, the present study is the first to demonstrate that lncRNA799 is an indicator of prognosis in cervical cancer. lncRNA799 levels were found to be correlated to tumor metastasis and poor prognosis. With regard to its mechanism of action, it seems that lncRNA799 reduces the inhibitory effect of miR-454-3p on TBL1XR1 by acting as a sponge for miR-454-3p (Figure 6E). The findings indicate that the ceRNA pathway involving lncRNA799 should be investigated for its role in cervical cancer progression.

## Materials and Methods

### RNA Isolation

RNA was isolated from the 218 cervical cancer tumors and adjacent normal tissues using TRIzol reagent (Invitrogen) according to the manufacturer’s instructions. The clinical characteristics of the patients are listed in Table 1. The study was approved by the medical ethics committee of The Second Affiliated Hospital of Nanchang University.

### Real-Time PCR

Target gene expression was analyzed by real-time PCR using the SYBR Green qRT-PCR kit (Takara, Dalian, China) according to the manufacturer’s instructions. The following primers were used: lncRNA799(MG201855), 5′-CAGGCAGGTGGCAGGTTTG-3′ (sense) and 5′-AGTGGGAGTCTGTGGATGAGC-3′ (antisense); transducin β-like protein 1-related protein (TBL1XR1), 5′-CTAGCACCTTAGGGCAGCATAAAG-3′ (sense) and 5′-GTCTTGTCTACTCCAGCACTTAGG-3′ (antisense). The reaction conditions were as follows: 95°C for 30 s, 40 cycles of denaturation at 95°C for 5 s, annealing at 60°C for 30 s, and extension at 60°C for 30 s. The flounder GAPDH gene was used as a housekeeping reference gene to normalize expression of the genes across samples. Fold changes in relative gene expression compared with the control were determined by the standard 2−ΔΔCt method.

### Cell Culture

The cervical cancer cell line (SiHa and C33a) was cultured in SH30243.01B medium (HyClone, USA) supplemented with 10% fetal bovine serum (FBS) (10099-141; GIBCO, Australia) and 1% penicillin and streptomycin (SV30010; HyClone, USA). Cells were grown at 37°C in an atmosphere containing 5% CO_2_.

### Plasmid Construction

An lncRNA799 expression plasmid was constructed by inserting lncRNA799 cDNA into the pReceiver plasmid (pCDNA3.1) (Wuhan GeneCreate Biological Engineering) according to the kit protocol. The constructed plasmid was transfected into SiHa cells using Lipofectamine 2000 (Invitrogen, CA, USA) according to the kit protocol. For lncRNA799 knockdown, a human short hairpin (sh) RNA sequence was cloned into the pSuper-retro-puro plasmid to create pSuper-retro-lncRNA799-RNAi(s) with the following sequence: 5ʹ-GATCAAGAGAATTAAAGCCATCCTTCAAGAGAGGATGGCTTTAATTCTCTTTTTTAATT-3ʹ. Stable cell lines expressing lncRNA799 shRNAs were selected and treated for 10 days with 0.5 mg/mL puromycin (Wuhan GeneCreate Biological Engineering).

### siRNA Transfection

Cells of the cervical cancer cell line SiHa were transfected with 25 nM non-targeting control siRNA or synthetic siRNAs (Wuhan GeneCreate Biological Engineering) against lncRNA799 (si-lnc799#1: 5′-GGAUGGCUUUAAUUCUCUUTT-3′ and 5′-AAGAGAAUUAAAGCCAUCCTT-3′; si-lnc799#2: 5′-CCACUUCUCUUGCCCAUAUTT-3′ and 5′-AUAUGGGCAAGAGAAGUGGTT-3′) for 48 hr using the Lipofectamine transfection reagent (Invitrogen). The cells were then collected, and lncRNA799 knockdown was confirmed by qRT-PCR. siRNAs against TBL1XR1 were generated as previously described.^7^

### Wound Healing Assay

After 12 hr of incubation, confluent monolayers that were formed on 35-mm Petri dishes were washed with PBS. A wound was created using a sterile pipette tip. Wound healing was evaluated under a phase-contrast microscope and photographed at ×200 magnification under a Zeiss microscope (Germany) at 0, 24, and 48 hr after the wound was made.

### Invasion Assay

Matrigel invasion assays were conducted using Invasion Chamber (Becton Dickinson, Bedford, MA, USA), according to the protocol of the manufacturer. In brief, cells were resuspended in serum-free medium at a density of 1 × 10^5^ cells/mL. The cells were then seeded into Transwell filters (pore size, 8 μm) coated with Matrigel. In the bottom chamber, medium containing 10% FBS was placed. Both of the chambers were then incubated at 37°C for 24 hr. After incubation, the filters were extracted and fixed, following which 0.05% (v/w) crystal violet staining was performed. The number of cells that had invaded was counted under a microscope; 10 high-power fields were randomly selected for each filter.

### Western Blot Assay

Western blot assays were performed as previously described,^7^ with anti-TBL1XR1 and anti-β-actin antibodies diluted to 1:1,000 (Proteintech, Wuhan, China). β-Actin was used as an internal control.

### Luciferase Activity Assay

Luciferase assays were performed using the Dual Luciferase Reporter Gene Assay Kit (Beyo-time Biotech Institute) according to the manufacturer’s instructions, and results were expressed as the ratio of luciferase to pRL-TK (mean ± SEM).

### RNA-Binding Protein Immunoprecipitation

miR-454-3p was transfected into SiHa cells. The RIP kit was purchased from Magna (catalog no. 17-707). RNA immunoprecipitation with a monoclonal anti-Ago2 antibody was used to assess endogenous Ago2 binding to RNA; IgG was used as the control. The levels of lnc799 and miR-454-3p were determined by qRT-PCR and presented as fold enrichment in Ago2 relative to input.

### Statistical Analysis

The SPSS 16.0 software (SPSS, Chicago, IL, USA) was used for statistical analysis. p < 0.05 was considered to indicate statistical significance.

## Author Contributions

L.-M.L. and F.-H.Z. participated in the study design and experimental work. G.-J.Y. and S.-F.A. participated in sample collection and data analysis. M.Z. participated in sample collection and conducted the experiments. L.H. conceived the study, was responsible for its design and coordination, and participated in the analysis and interpretation of the data, as well as drafting and revising all versions of the manuscript.

## Conflicts of Interest

The authors have no competing interests.
